# Release, Reentry, and Reintegration During COVID-19: Perspectives of Individuals Recently Released from the Federal Bureau of Prisons

**DOI:** 10.1089/heq.2022.0172

**Published:** 2023-07-10

**Authors:** Camille Kramer, Minna Song, Carolyn B. Sufrin, Gabriel B. Eber, Leonard S. Rubenstein, Brendan Saloner

**Affiliations:** ^1^ Department of Gynecology and Obstetrics, Johns Hopkins University School of Medicine, Baltimore, Maryland, USA.; ^2^ Department of Health Policy and Management, Johns Hopkins Bloomberg School of Public Health, Baltimore, Maryland, USA.; ^3^ Department of Health, Behavior and Society, and Johns Hopkins Bloomberg School of Public Health, Baltimore, Maryland, USA.; ^4^ Department of Epidemiology, Johns Hopkins Bloomberg School of Public Health, Baltimore, Maryland, USA.

**Keywords:** reentry, COVID-19, Federal Bureau of Prisons, depopulation, incarceration

## Abstract

**Introduction:**

The COVID-19 pandemic had a large negative impact on people in U.S. prisons. Expedited releases from prison were one strategy used to decrease morbidity and mortality from COVID-19. However, little is known about the reentry experiences of those being rapidly released from custody early in the pandemic.

**Methods:**

We aimed to examine the perspectives of former residents in the Federal Bureau of Prisons (BOP) regarding release, reentry, and reintegration into their respective communities. We conducted semistructured interviews with 21 recently released individuals primarily recruited through legal aid organizations between September and October 2021. Subjects were incarcerated before and during the early surge in the COVID-19 pandemic. We coded transcripts thematically with domains developed *a priori* in which we revised iteratively and inductively based on the data.

**Results:**

Several major themes emerged. Participants reported that they needed to advocate for themselves to take advantage of the early release process. Compared with normal circumstances, they reported a lack of reentry planning and preparation before participants were released. Finally, experiences with reintegration varied but were often more challenging due to COVID-19.

**Discussion:**

Residents released during COVID-19 reported many challenges with reentry that could have been mitigated by support and guidance from the BOP. Reentry is a process that should begin prelease and continues postrelease to ensure individuals have adequate structural and social supports.

**Health Equity Implications:**

Inadequate reentry support has significant impacts on the health and well-being of recently released individuals and contributes to the broader context of achieving health equity for minitorized groups who are disproportionately overrepresented in prisons. Policy and practice reform is needed to address the time-sensitive, life-threatening challenges individuals face when transitioning from prison to community.

## Introduction

The COVID-19 pandemic has had a substantial impact on the health and safety of people incarcerated in the United States, with much higher rates of cases and deaths in prisons than in the general population.^1^ Reduction in the prison population through expedited releases became an important strategy to mitigating the impact of COVID-19 on incarcerated individuals. Due to systemic racism and systems of oppression, socially marginalized groups are overrepresented in prisons. This includes minoritized populations, individuals with stigmatized behavioral health conditions such as mental illness and substance use disorders, and those who often have inadequate access to health care in the community and underlying health conditions.^2^ Thus, the rapid release of individuals due to COVID-19 has implications for both the safety and well-being of those in custody as well as in addressing health equity.

However, the reentry experiences of individuals rapidly released during the pandemic have not been well-documented. Studies indicate that individuals returning to their communities struggle with meeting their basic needs such as housing, employment, food, and health care, as well as with social integration and connectedness.^3–5^ Prison to community transitions encompass reestablishment of relationships with peers and family members and avoidance of technical violations that can result in reincarceration,^6,7^ yet many community services that provide reentry support may have been curtailed during the pandemic, particularly in the early months.^8,9^

The Federal Bureau of Prisons (BOP) is the largest carceral system in the United States and primarily houses people who are detained pretrial, or have been convicted, for federal offenses. To respond to the growing concern about COVID-19 in prisons, the Coronavirus Aid, Relief, and Economic Security (CARES) Act released thousands of BOP residents to home confinement. Under ordinary circumstances, the BOP prioritizes reentry planning, particularly during the 18 months before release.^10^ This planning includes a curriculum focused on securing employment after release (e.g., resume writing, job searching, and job retention) and getting acclimated to life in the community. Additional available programming includes literacy classes,^11^ wellness education, parenting classes, and substance use treatment,^12^ as well as vocational training. However, it is unclear what, if any, reentry supports were provided to people released on the expedited timetable that characterized COVID-19-related releases.

In this study, we focus on the release, reentry, and reintegration experiences of people released from Federal BOP facilities during COVID-19. We provide perspectives from recently released individuals from BOP facilities during the COVID-19 pandemic, including their accounts navigating release in their facilities, reentry planning or the lack thereof, and experiences of return to the community.

## Materials and Methods

This study involved semistructured, qualitative phone interviews with a sample of previously incarcerated BOP residents in custody before March 2020 and released from custody during the COVID-19 pandemic.

We recruited individuals through referrals by public defenders and publicly appointed attorneys. Study recruitment and interviews were conducted between August and October 2021. We scheduled interviews at least 2 days after the recruitment call to allow people to fully consider their participation. Interviews lasted ∼1 h and were audio-recorded on Google Voice, then professionally transcribed. Participant remuneration was a $50 gift card. Eligibility criteria included being at least 18 years of age, English-speaking, and having both pre-COVID-19 and during-COVID-19 incarceration experience. We did not disclose the identities of study participants to the referral source or the BOP.

The semistructured interview guide explored participant perspectives on being incarcerated during COVID-19. The interviews were part of a larger project focused on fairness and justice in health care resource allocation for incarcerated people during the COVID-19 pandemic. The broader study was grounded in human rights and ethics theory,^13^ and we designed questions to elicit reflections on aspects of being incarcerated during COVID-19 that challenged the fulfillment of basic rights. We developed questions to elicit reflections about equitable treatment and fair process grounded in principles of human rights^14^ and focused on the response of facilities to health and safety needs of residents during the COVID-19 pandemic.

One section focused on perceptions related to transitioning out of custody during the pandemic, including navigating the release process, what BOP did to aid in release and reentry, and the experience of reintegrating back to their home communities. Specifically, we asked participants whether they perceived that the timing of their release (or others) was impacted by COVID-19, how life has been on the outside since release, what were the most difficult aspects about being released, and how the prison could improve the experience of those being released during COVID-19. We used nonstigmatizing language such as “residents” instead of “inmates” in the interviews, and in this article, when referring to people who are incarcerated.^15,16^

Two research team members (C.K. and M.S.) coded the interview transcripts using Dedoose software,^17^ guided by a directed content analysis approach.^18^ We developed *a priori* domains for analysis in the planning stages of the study, and then revised codes iteratively and inductively based on data captured in the interviews. Two exemplary transcripts were coded by both coders who met regularly until consensus was reached. The remaining transcripts were split and single-coded by the two coders who continued to meet weekly to ensure agreement in code application.

Our primary analytical goal was to capture the subjective reentry experience of our participants. Accordingly, we adopted an interpretivist epistemological perspective to what participants reported, acknowledging that the realities experienced by these individuals may not match those reported by officials in the system or other observers. When individuals reported, for example, beliefs about the ulterior motives of carceral staff, we report their beliefs because they inform the worldview or perspective of the participants. We do not, however, attempt to adjudicate the underlying facts of the situations.

We followed standards for conducting and reporting qualitative research.^19^ We stopped recruitment once thematic saturation was reached based upon similar accounts reported by participants. We verified the validity of the data by triangulating our findings with reports regarding release challenges and the nuances of reentry with existing literature both before COVID-19 and reports during the pandemic. We supported our findings through empirical data (i.e., participant quotes) from the interview transcripts. We addressed the limitations of our study and findings to promote research transparency.

The Johns Hopkins University Bloomberg School of Public Health Institutional Review Board approved this study. All participants consented to be in the study.

## Results

Twenty-one former BOP residents completed a qualitative interview. Most participants were men (*N*=15), had a least a high school diploma or general education degree (*N*=20), and reported public health insurance postrelease (*N*=8 Medicaid, *N*=4 Medicare, *N*=6 private insurance, *N*=3 no insurance) (Table 1). Participants' racial identities were mostly split between white (*N*=9) and black (*N*=8), with one person identifying as Asian, Native Hawaiian, or Pacific Islander. Three participants identified as Hispanic, their race was unknown. The average age of participants was 53, which is considered geriatric for people who are incarcerated,^20^ with a range of 31–76. Twelve participants self-reported a chronic illness (e.g., cancer, chronic obstructive pulmonary disease, asthma, and colitis) for which they received medical care while being incarcerated.

**Table 1.** Participant demographics and other characteristics

	*N* =21
Gender	
Man	15
Woman	5
Transgender	1
Race	
White	9
Black, African American	8
Asian, Native Hawaiian, or Pacific Islander	1
American Indian or Alaskan Native	0
Unknown	3
Ethnicity	
Hispanic	3
Non-Hispanic	18
Age	
31–40	3
41–50	8
51–60	4
61–70	4
71–80	2
Highest education level	
Some high school	1
High school diploma/GED	11
Some college (or trade school)	4
College degree (bachelor's, associate)	4
Graduate degree (master's, doctorate)	1
Region of residence preincarceration	
Northeast	2
Midwest	7
South	4
West	8
Region of incarceration facility^a^	
Northeast	3
Midwest	4
South	9
West	9
Health insurance coverage postrelease	
No insurance	3
Private	6
Public	12
Medicare	4
Medicaid	8
Length of stay, years	
0–10	11
11–20	6
21–30	1
31–40	1
40–50	1
Unknown	1
COVID-19 infection while incarcerated	
Yes	9
No	10
Suspected (i.e., had symptoms but was not tested)	2

^a^ Three participants were at more than one facility during the COVID-19 pandemic

GED, general education degree.

Years of current incarceration ranged from 2 to 44 years, with most being incarcerated fewer than 10 years (*N*=11). Individuals were released between April 2020 and August 2021, with a median release date of February 2021. Time since release varied, with an average of 8 months (range of 1–17 months) out of custody at the time of the interview. Participants identified varying pathways for release including the CARES Act, compassionate release, the Second Chance Act, and parole. All participants reported being under correctional supervision (e.g., parole, probation, or home confinement) at the time of the interview.

We identified three key themes regarding participants' experiences of release, reentry, and reintegration during COVID-19: (1) participants' agency in navigating their release with reportedly little to no help; (2) according to participants little to no reentry planning and preparation for release were provided; and (3) participants' reported having mixed experiences with reintegration after release.

### Participants' agency in navigating their release with reportedly little to no help

*(1a) Participants reported learning of early release opportunities through various means—from the warden in the facility who encouraged release applications, from advocacy organizations that sent information to people in custody, or by word of mouth from other residents* [Table 2, quotes 1–3]. According to participants, navigating release meant determining which mechanism of release best suited their situation, locating and filling out the necessary paperwork that included reporting where they would be living and receiving medical care if they have a chronic illness, and filing the motion with the court. Most participants expressed doing this with no help from facility staff during the pandemic, when many systems were shut down or delayed, despite BOP's declared urgency in releasing individuals.^21^
Some reported receiving assistance from their lawyers. One participant mentioned that her network of people in the community helped advocate for her release by bringing attention to the prison. She was one of only two participants who noted that a staff member came to them to inform them that they may qualify for early release.
*(1b) Some participants demonstrated self-efficacy for release despite the belief that their self-agency was thwarted by staff who they viewed as barriers in the release process* [Table 2, quotes 4–6]. Some interviewees described logistical and structural obstacles to release and thwarted attempts of residents' self-advocacy. One reported that his case manager was not aware that he was being released until just hours before he left the facility.
*(1c) BOP's limited response to assisting with the release of people in custody, as reported by our participants, led some to believe that staff did not want to release residents* [Table 2, quotes 7–11]. Some residents believed that there was an ulterior motive to prevent releases such as financial gain or as a means for the prison to keep receiving federal funds. One respondent, a 47-year-old male participant, said that facility staff were not happy he was leaving and said he overheard the warden saying to the person processing his release that they “need to get more people in” to offset the residents who are leaving the facility on early release. Another believed that keeping people in custody meant job security for case workers and security of federal dollars.
Some said that facility leadership denied applications and expressed sentiments that residents would be safer from COVID-19 if incarcerated, which led residents to believe that staff wanted to keep them in custody.

### According to participants, little to no reentry planning and preparation for release were provided

Almost all of the participants in our sample reported being released from custody earlier than their sentence end date, sometimes years earlier. One participant, a 42-year-old male participant, described how the first few months were stressful because the arrangements he made were for the future. “I was out two years early. So all the plans that I had made—and my family had made—were for two years in the future.” Interviewees reported that they were grateful to be released early, but faced navigating the reentry process with little assistance from the BOP. Participants described being given a trash bag and told to gather their belongings, sometimes just hours before their release, and being left at the airport with money sufficient to buy only a day's worth of food. Some shared they were not even able to alert their family members when they would arrive home.

**Table 2.** Representative quotes for emergent themes

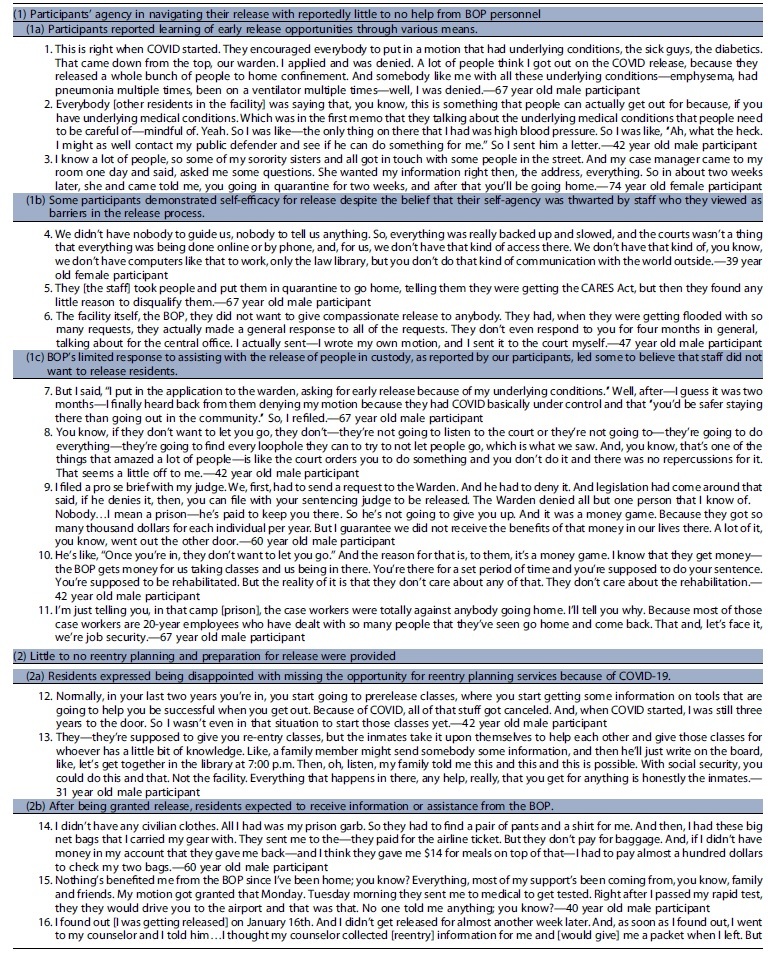 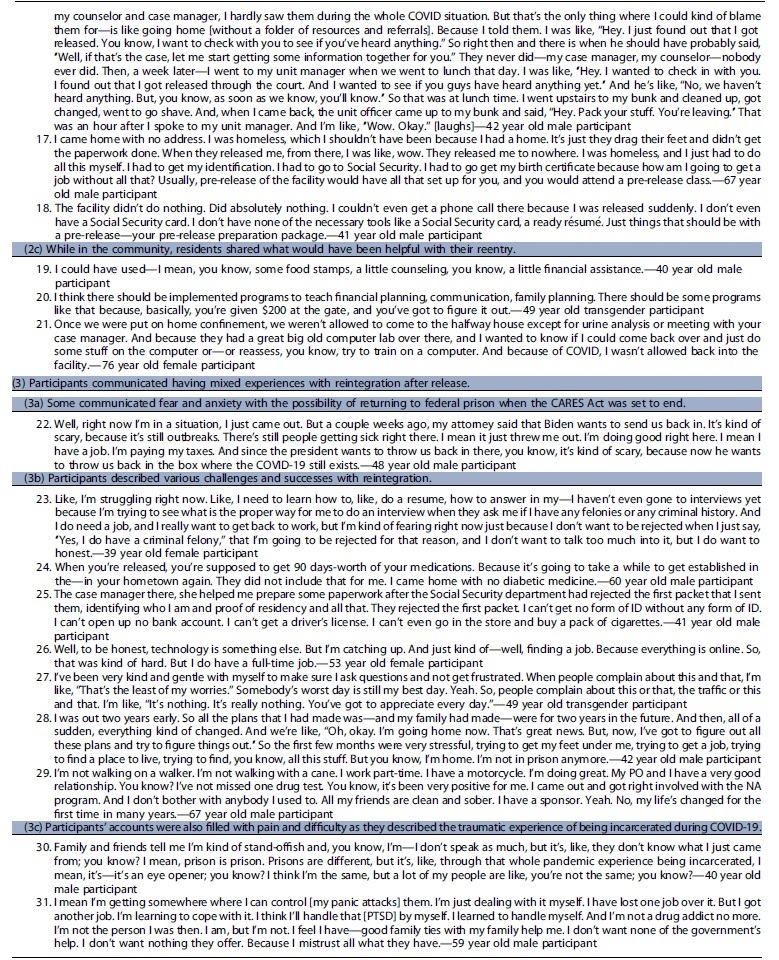 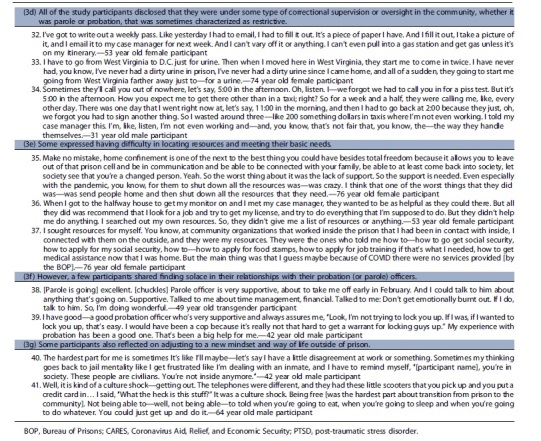

Notably, almost all participants were incarcerated in states other than their state of residence before imprisonment. When asked what the BOP did to aid or facilitate the transition from prison to the community, an overwhelming number of participants said “nothing.” As a 59-year-old male participant stated, “[they did] zero. No financial help. No medical help. No psychiatry. Nothing. There's the door. Get out. We don't want to see you again.”

*(2a) Residents expressed being disappointed with missing the opportunity for reentry planning services because of COVID-19* [Table 2, quote 12–13]. Some reflected on what the BOP did for reentry pre-COVID, such as classes that begin 2 years before release to prepare residents for life in the community. According to BOP public materials, reentry classes include programming on how to secure employment and get acclimated back into the community with additional classes focusing on literacy, parenting skills, and wellness education.^10^ Residents expressed disappointment with missing the opportunity for reentry programming due to COVID-19. They communicated that the absence of reentry classes greatly impacted their preparedness for life in the community and created additional stress. One mentioned how residents learned about reentry support through other means such as sharing the information from family and friends in the community.
*(2b) After being granted release, residents expected to receive information or assistance from the BOP* [Table 2, quotes 14–18]. Despite the lack of programming, most participants thought they would at least receive a folder upon release containing all the relevant paperwork, information (i.e., social security card, license), and other resources that would aid with the reentry process. However, they reported receiving nothing. *(2c) While in the community, residents shared what would have been helpful with their reentry* [Table 2, quotes 19–21]. Once back in the community, some participants reflected on what would have been helpful with their reentry including things such as financial and other assistance, behavioral health support, family planning resources, and technology classes.

### Participants communicated having mixed experiences with reintegration after release

*(3a) Some communicated fear and anxiety with the possibility of returning to federal prison when the CARES Act was set to end (before the December 21, 2021 statement from the Attorney General that clarified that those released under the CARES Act would not have to return to prison once the threat of COVID-19 ends)* [Table 2, quote 22].^22^ These fears and concerns were compounded by worries about returning to prison and being subjected to the poor conditions and mistreatment they experienced due to the pandemic.
*(3b) Participants described various challenges and successes with reintegration* [Table 2, quotes 23–29]. Many shared that they were able to successfully secure employment, reconnect with their family and friends, and meet their basic needs. Despite these successes, some identified specific obstacles, highlighting how bureaucracy created challenges—navigating disclosure of their incarceration history in job interviews, obtaining diabetic medications or health insurance, which was often delayed because the BOP did not provide bridge medications, trying to locate one's social security card when many governmental agencies had limited services due to the pandemic, and getting acclimated to the use of new technology such as virtual conferencing.
*(3c) Participants' accounts were also filled with pain and difficulty as they described about the traumatic experience of being incarcerated during COVID-19* [Table 2, quotes 30–31]. Some participants described the ongoing trauma of incarceration during the height of COVID-19 and sequelae such as nightmares, anxiety attacks while in crowded spaces, and navigating difficult social dynamics such as family expectations that they be the same people they were preincarceration.
*(3d) All of the study participants disclosed that they were under some type of correctional supervision or oversight in the community, whether it was parole or probation, that was sometimes characterized as restrictive* [Table 2, quotes 32–34]. They believed that these intermediate sanctions^23^ made it difficult to fulfill their daily tasks or required them to navigate obstacles to avoid violating the terms of their release. For instance, one participant described having to spend hundreds of dollars on taxi fares to comply with frequent and last-minute requests for urine drug screens ordered by his probation officer. Another, a 76-year-old female participant, described how she was reincarcerated for 3 weeks because the halfway house was unable to contact her while she was participating in an approved computer class that was offered by an outside organization due to COVID-19.
*(3e) Some expressed having difficulty in locating resources and meeting their basic needs* [Table 2, quotes 35–37]. For example, participants shared that their halfway houses required that they be employed but provided no help obtaining a job, a problem compounded by many social services being closed due to the pandemic. *(3f) However, a few participants shared finding solace in their relationships with their probation (or parole) officers* [Table 2, quotes 38–39]. They noted having helpful interactions and communicated that they could rely on these individuals to help them navigate any challenges they encountered. *(3g) Some participants also reflected on adjusting to a new mindset and way of life outside of prison* [Table 2, quotes 40–41].

## Discussion

Early releases from prison initiated during the COVID-19 pandemic have been considered a potential opportunity to advance the larger goal of curbing mass incarceration in the United States by reducing the overall size of the prison population. Study participants were released during a period of substantial stress in BOP facilities and when many social services on the outside were not fully operational.^24^ Their reported that self-efficacy, self-advocacy, and social supports from people in the community were significant factors in facilitating their early release and navigating life as they reentered society. Yet, participants perceived that their releases unfolded with unpredictable timing and poor coordination, making them especially vulnerable to harms during this period. For example, the stress of transitioning from prison to community with little support was amplified for those who had to manage their chronic illness on their own in the community.

Access to primary care may reduce recidivism after release, but factors such as low rates of Medicaid enrollment among recently incarcerated impede this goal.^25,26^ Furthermore, there is robust literature demonstrating that people with incarceration histories have higher rates of experiences of trauma preincarceration and that incarceration itself is a traumatic experience.^27–30^ Relatedly, many participants described being incarcerated during COVID-19 as a uniquely traumatic experience, one that still affected them postrelease, according to participants. Reentry services including but not limited to behavioral health counseling should be attuned to the unique mental and physical trauma residents endured.

While many participants experienced major challenges upon return to the community, there were also notable successes. Despite the adversity they faced, similar to the findings of other studies,^4,6,7^ participants demonstrated resiliency and expressed joy and happiness for being released. These self-reported data are consistent with recent reports that indicate that the recidivism rate of people released from BOP under provisions including the CARES Act was exceedingly low—reported to be only 17 individuals out of 11,000 by September 2022.^31^

Community-based reentry support is still recovering from disrupted services due to the pandemic, with some services still not fully operational or have stopped operating altogether.^24^ This is especially troubling as community reentry service providers report that the community need is greater and that people need more support than they did prepandemic. Furthermore, our study shows that the consequences of incarceration during the most dire times of the pandemic last far beyond the period of immediate stabilization in community life. Trauma and the usual challenges associated with reentry require the attention of social service agencies for extended periods of time to match the ongoing challenges faced by those released. This finding may become particularly salient as members of the larger released population who were infected with COVID-19 experience the long-term health consequences of the virus that are only now beginning to be understood.

Our study has important policy implications. Systemic policy and practice changes are needed to improve the experiences of individuals being rapidly released from custody during a global pandemic. Several of the challenges anticipated by the National Academies of Sciences, Engineering and Medicine (NASEM) were anticipated by our respondents, including clear emphasis on safety during reentry. NASEM provides guidance on safe reentry and return addressing existing challenges with reentry (e.g., limited housing), also taking into account the impact of COVID-19 (e.g., shortage of reliable, safe noncongregate housing).^32^

They provide a list of specific action items carceral facilities can do to facilitate a smooth transition from prison to community such as providing residents with sufficient information before release that includes referrals and linkages to assistance programs and support services that meet their specific needs, and supplying recently released individuals with prepaid phones with video access so that they can contact community organizations that may be working remotely due to COVID-19. In addition to implementing the recommended practice changes, our research supports that there is also value in developing clearly delineated policy reform proposed by the NASEM including facilitating expedited Medicaid/Medicare, Supplemental Nutrition Assistance Program, and Supplemental Security Income enrollment prerelease and improving community health care access by removing requirements for government identification at the first visit for those recently released from custody.^32^

Other entities have acknowledged the reentry crisis, including the Reentry Coordination Council who produced a report to recognize and reduce reentry barriers informed by lessons learned from COVID-19.^33^ However, there is still value in developing operational plans for how any future expedited release would be coordinated, including plans to offer reentry programs on a compressed timetable and strategies for providing more intensive services in the weeks after release.

Several limitations should be considered when interpreting our study findings. First, our study is limited to individuals released from the BOP, the largest, but one of only hundreds of carceral systems in the United States, each of which is unique. Our findings cannot be generalized to other prisons, jails, or carceral systems, or to BOP facilities in which no study participants were incarcerated. Everyone in our sample was referred by their public defender or publicly appointed attorney, which may limit our sample to those who had better relationships with their lawyers or whose lawyers were more invested in their cases. One analytic limitation that could have great health equity implications was that although we had a diverse sample, we were not able to make explicit race comparisons and we did not probe for race-related experiences. More than half of our sample identified as black or Hispanic.

Literature supports that minorities face specific obstacles to reentry involving probation and parole, resource access, familial ties, economic and educational opportunities, community disadvantages, civic disenfranchisement, and access to health care.^34–36^ Future analyses should assess for any differences in reported experiences based on race and gender. In addition, our sample may be skewed in that we interviewed individuals who are older than the average for the prison population.

### Health equity implications

The pandemic provides an opportunity to reflect not only on issues of reentry, but also on the broader and related topics of mass incarceration and health and social equity. The experiences of people released from the federal prison population during the COVID-19 pandemic reveal some of the ways in which release from custody, an important step taken to protect people from COVID-19 and end unnecessary incarceration, nevertheless left them vulnerable to and forced them to adapt to novel and unexpected hardships as well as the well-established challenges of reentry.

In particular, this study demonstrated that while the release of people from custody is a step toward achieving health equity related to disproportionate policing and mass incarceration, it highlighted the need to address other social issues that contribute to health inequities such as inadequate access to health care, enhanced mental health and substance use disorder treatment, state and national policy reform regarding assistance and aid to individuals with a history of criminal legal involvement, and ultimately questioned the justification of keeping people in custody who, as demonstrated, can return to the community. It is critically important to engage with this population on a continuing basis to identify emerging challenges, to address social determinants of health, and to offer services that promote health and social equity.

## Abbreviations Used

BOP: Bureau of Prisons
CARES: Coronavirus Aid, Relief, and Economic Security
GED: general education degree
NASEM: National Academies of Sciences, Engineering and Medicine
PTSD: post-traumatic stress disorder
